# Bioactive Co(II), Ni(II), and Cu(II) Complexes Containing a Tridentate Sulfathiazole-Based (*ONN*) Schiff Base

**DOI:** 10.3390/molecules26103062

**Published:** 2021-05-20

**Authors:** Aurora Reiss, Nicoleta Cioateră, Aurelian Dobrițescu, Mihaela Rotaru, Alice Carla Carabet, Filippo Parisi, Anca Gănescu, Irina Dăbuleanu, Cezar Ionuț Spînu, Petre Rotaru

**Affiliations:** 1 Department of Chemistry, Faculty of Sciences, University of Craiova, 13 AI Cuza Street, 200585 Craiova, Romania; reissaurora@yahoo.com (A.R.); nicoletacioatera@yahoo.com (N.C.); dobri_aur@yahoo.com (A.D.); anca_ganescu@yahoo.com (A.G.); irinanicolaescu@yahoo.fr (I.D.); spinu_cezar@yahoo.com (C.I.S.); 2 Institute of Chemistry of the Academy of Sciences of Moldova, 3 Academiei Street, MD2028 Chișinău, Moldova; rmihaela1993@gmail.com; 3 Department of Physics, Faculty of Sciences, University of Craiova, 13 AI Cuza Street, 200585 Craiova, Romania; alicecarla.benis@gmail.com (A.C.C.); petrerotaru@yahoo.com (P.R.); 4 Department of Physics and Chemistry Emilio Segrè, University of Palermo, Viale delle Scienze, Ed. 17, I-90128 Palermo, Italy; 5 Department of Mathematics and Geosciences, University of Trieste, Via Weiss 2, I-34128 Trieste, Italy

**Keywords:** Schiff base, sulfathiazole, Co(II), Ni(II) and Cu(II) complexes, thermal behavior, antibacterial activity

## Abstract

New Co(II), Ni(II), and Cu(II) complexes were synthesized with the Schiff base ligand obtained by the condensation of sulfathiazole with salicylaldehyde. Their characterization was performed by elemental analysis, molar conductance, spectroscopic techniques (IR, diffuse reflectance and UV–Vis–NIR), magnetic moments, thermal analysis, and calorimetry (thermogravimetry/derivative thermogravimetry/differential scanning calorimetry), while their morphological and crystal systems were explained on the basis of powder X-ray diffraction results. The IR data indicated that the Schiff base ligand is tridentate coordinated to the metallic ion with two N atoms from azomethine group and thiazole ring and one O atom from phenolic group. The composition of the complexes was found to be of the [ML_2_]∙nH_2_O (M = Co, *n* = 1.5 (1); M = Ni, *n* = 1 (2); M = Cu, *n* = 4.5 (3)) type, having an octahedral geometry for the Co(II) and Ni(II) complexes and a tetragonally distorted octahedral geometry for the Cu(II) complex. The presence of lattice water molecules was confirmed by thermal analysis. XRD analysis evidenced the polycrystalline nature of the powders, with a monoclinic structure. The unit cell volume of the complexes was found to increase in the order of (2) < (1) < (3). SEM evidenced hard agglomerates with micrometric-range sizes for all the investigated samples (ligand and complexes). EDS analysis showed that the N:S and N:M atomic ratios were close to the theoretical ones (1.5 and 6.0, respectively). The geometric and electronic structures of the Schiff base ligand 4-((2-hydroxybenzylidene) amino)-*N*-(thiazol-2-yl) benzenesulfonamide (HL) was computationally investigated by the density functional theory (DFT) method. The predictive molecular properties of the chemical reactivity of the HL and Cu(II) complex were determined by a DFT calculation. The Schiff base and its metal complexes were tested against some bacterial strains (*Escherichia coli*, *Pseudomonas aeruginosa*, *Staphylococcus aureus*, and *Bacillus subtilis*). The results indicated that the antibacterial activity of all metal complexes is better than that of the Schiff base.

## 1. Introduction

Sulfathiazole, 4-amino-*N*-(1,3-thiazol-2-yl) benzenesulfonamide, is a short-acting sulfa drug that, in the past, was used as an oral antimicrobial until other less toxic sulfonamides antibiotics were discovered [1]. In the last few years, interest in transition metal complexes with Schiff bases derived from sulfonamide antibiotics, also called sulfa drugs, has increased due to their potential applications in medicine, chemistry, and biology [2,3,4,5,6,7,8,9,10,11,12]. The coordination of Schiff bases derived from sulfonamides with transition metal ions improves the bases’ biological activity. In order to explain this behavior, it is necessary to understand the relationship between metals and ligands in biological systems, as well as the ease of cleaving the bond between the metal ion and the ligand in these systems. In this context, seven binuclear Cr(III), Fe(III), Cu(II), ZrO(II), Sn(II), Pb(II), and Ce(III) complexes with the Schiff base obtained via condensation between 2-(4-aminobenzenesulfonamido)thiazole and 2-hydroxybenzaldehyde were prepared, characterized, and tested for antitumor and antimicrobial efficiencies [13]. Cephalexin, a first generation cephalosporin, was used to obtain a Schiff base ligand following the reaction with sulfathiazole. Mn(II), Co(II), Ni(II), Cu(II), and Zn(II) complexes with this Schiff base were synthesized, characterized by various techniques, and tested for antibacterial activity. The results of the biological tests showed that the Cu(II) and Zn(II) complexes presented higher activity than the Mn(II), Co(II), and Ni(II) complexes [14].

Following our earlier work in searching for new bioactive metal complexes with Schiff bases derived from drugs [15,16,17,18], the present paper deals with the synthesis, physico-chemical characterization, and investigation of the thermal behavior of new Co(II), Ni(II), and Cu(II) complexes with the Schiff base 4-((2-hydroxybenzylidene)amino)-*N*-(thiazol-2-yl)benzenesulfonamide (HL) derived from the condensation of a sulpha drug, sulfathiazole (Stz), and salicylaldehyde [19]. We selected the three metal ions to obtain complexes based on this Schiff base because (i) they had not been used for this purpose so far and (ii) they have often been studied when it comes to synthesizing complexes with drugs/Schiff base with drugs to be tested for biological activity. Two Gram-negative (*Escherichia coli* and *Pseudomonas aeruginosa*) and two Gram-positive (*Staphylococcus aureus* and *Bacillus subtilis*) bacterial strains were used for testing the Schiff base and its metal complexes regarding their in vitro antibacterial activity [15,16,20,21].

## 2. Experimental and Theoretical Premises

### 2.1. Materials

All used chemicals and solvents were of analytical grade. Sulfathiazole, CoCl_2_·6H_2_O, NiCl_2_·6H_2_O, CuCl_2_·2H_2_O, and salicylaldehyde were from Sigma-Aldrich.

### 2.2. Equipments and Characterization Techniques

An M.L.W. CHN analyzer was used for the chemical analyses of carbon, hydrogen, and nitrogen. A Varian-AA775 spectrophotometer was used to determine the metal contents of the complexes using the atomic absorption technique. A Perkin Elmer 157 instrument in anhydrous KBr pellets recorded IR spectra in the range of 4000–400 cm^−1^, and a Jasco V670 spectrophotometer with MgO as the standard recorded electronic spectra by the diffuse reflectance technique (DRUV) in the range of 5000–50,000 cm^−1^. A Sanyo Gallen Kamp apparatus was used for the melting points, and an OK-102 conductivity meter was used for determining the molar conductivities. NMR spectra were recorded on a Bruker Ultra shield 400 Plus. A Faraday balance that works at room temperature was used for magnetic susceptibility measurements.

A horizontal DIAMOND TG/DTA analyzer from PerkinElmer Instruments was used for thermal analysis measurements (TG/DTG/DTA/DSC) in a dynamic air atmosphere (150 cm^3^ min^−1^) under non-isothermal linear regimes [21,22,23,24,25,26,27,28]. Samples from 1.35 to 3.69 mg in alumina crucibles were heated in air in the range from RT to 1000 °C at a heating rate of 10 °C min^−1^.

A Hitachi SU8010 scanning electron microscope was used to perform the morphological examinations of the polycrystalline powders. An Oxford EDXS unit that was built into the microscope and capable of determining elements down to Be was used for the point elemental analysis of the samples. A SmartLab diffractometer from Rigaku in the range of 2θ = 10–80° with a 0.02° step using CuKα radiation was used to record the X-ray diffraction patterns of the polycrystalline powders. The ICDD database was used to perform phase identification. The FullProf software was used to refine the unit cell parameters by pattern matching [29].

### 2.3. Antibacterial Activity Study

The in vitro biological screening effects of the compounds were tested against two Gram-negative (*Escherichia coli* and *Pseudomonas aeruginosa*) and two Gram-positive (*Staphylococcus aureus* and *Bacillus subtilis*) clinical isolate bacterial strains via the well diffusion method using agar nutrient as the medium and amoxicillin as the control. According to the common procedure used in hospitals, the diffusion method was done as follows: on an agar plate inoculated with bacterial strains, a well was made and filled with the test solution with a micropipette. The prepared plate was incubated at 30 °C for 72 h, during which the test solution diffused and the growth of the inoculated bacterial strains was influenced. Then, the developed inhibition zone was measured. The activities of the Schiff base ligand and its metal complexes were confirmed by calculating the activity index (AI) [30].

### 2.4. Computational Details

A 3D molecular model of the copper complex in the fundamental state of the doublet was obtained from a previous geometric optimization calculation through a method of molecular mechanics based on force fields. The geometric preoptimization calculation was followed by a density functional theory (DFT)-type calculation of the molecular structure.

DFT calculations of the geometry of the ligand molecule in the singlet ground state and of the geometry of the transition metal complex in the doublet ground state were performed on the basis of the hybrid model of the B3LYP (Becke 3 Lee Yang Parr) exchange-correlation function [31] using the basic set of Pople-type 6-31G (d, p) for all atoms (C, H, O, N, and S) except for the transition metal. In this case, the effective core potential double-ζ basis set, LANL2DZ, was used [32]. Calculations of the geometry of the molecule in the ground state of singlet, the electronic structure, and the total energy of the molecule were performed based on the hybrid model of the B3LYP exchange-correlation function using the basic set 6-31G (d, p). Geometric optimization without symmetry restriction was performed in Cartesian coordinates at the Restricted Hartree–Fock (RHF) level. The optimization calculation was done using Pulay DIIS (direct inversion in the iterative subspace) procedure, the optimization algorithm used being GDM (geometric direct minimization). Convergence acceleration was achieved by approaching the Broyden–Fletcher–Goldfarb–Shanno (BFGS) algorithm in the iterative subspace. The stationary point located on the potential energy surface was validated as a local minimum by calculating the frequencies corresponding to the vibration modes without obtaining any values belonging to the set of complex numbers: the calculated value of the Hessian index was zero.

### 2.5. Synthesis of the Schiff Base (HL)

The ligand was synthesized according to a method reported in literature but with some changes [19]. First, 1 mmol sulfathiazole in 30 mL of ethanol was added dropwise under continuous stirring to 1 mmol salicylaldehyde in 15 mL of ethanol, and the mixture was refluxed for 3 h. The obtained precipitate was filtered off, washed with ethanol, and dried under vacuum. The purified Schiff base was obtained by recrystallization in hot ethanol.

**Figure 1:**
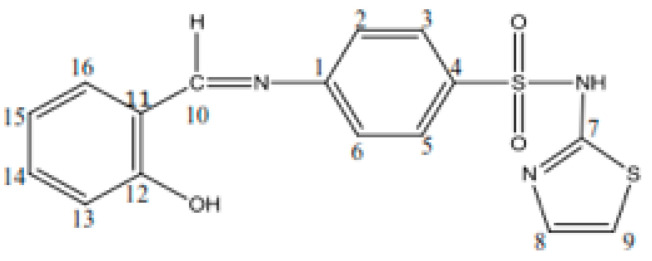
Structural formula with the atom numbering of the Schiff base (HL) ligand.

Schiff base (HL): C_16_H_13_S_2_O_3_N_3_, yield, %: 85, color: orange, m.p. 232–234 °C. Found (calcd.) %: C, 53.44 (53.48); H, 3.51 (3.62); N, 11.80 (11.70); ^1^H NMR (DMSO-d_6_, *δ*, ppm): ^1^H NMR (DMSO-d_6_, 400 MHz, *δ*, ppm): 7.28 (d, 1H, thiazole), 6.86 (d, 1H, thiazole), 7.52–7.88 (m, 4H, N-Ph), 12.8 (s, 1H, SO_2_NH-), 8.9 (s, 1H, azomethine), 12.6 (s, 1H, OH), 7.46 (s, 1H, -phenyl), 7.0 (s, 1H, -phenyl), 7.69 (s, 1H, -phenyl). ^13^C NMR (100 MHz, *δ*, ppm): 122.2 (C_2_, C_6_ N-Ph), 127.7 (C_3_, C_5_ N-Ph), 169.0 (C_7_ thiazole), 125.1 (C_8_ thiazole), 108.8 (C_9_ thiazole), 165.5 (C=N azomethine), 152.0 (C_1_ N-Ph), 140.7 (C_4_ N-Ph), 119.8 (C_11_, C_15_ OH-Ph), 160.7 (C_12_ OH-Ph), 117.2 (C_13_ OH-Ph), 134.3 (C_14_ OH-Ph), 133.0 (C_16_ OH-Ph). Figure 1 shows the structural formula of the Schiff base (HL) ligand with atom numbering.

### 2.6. Synthesis of the Metal Complexes

The three metal complexes (1)–(3) were prepared in the same way. A solution obtained by dissolving 1 mmol metal salt in 15 mL of ethanol was added under stirring to a hot solution of 2 mmol Schiff base in 40 mL of ethanol. The mixture was refluxed for 4 h. The volume of the mixture was reduced to half using a rotavapor when a colored product was obtained. The precipitate was separated by filtration, washed with ethanol and diethyl ether, and dried under vacuum over anhydrous CaCl_2_.

(1) CoC_32_H_27_S_4_O_7.5_N_6_, yield, %: 80, color: dark green. Found (calcd.) %: C, 47.28 (47.35); H, 3.37 (3.45); N, 10.06 (10.35); Co, 7.19 (7.26). *λ*_M_ = 18 Ω^−1^ cm^−2^ mol^−1^;
(2) NiC_32_H_26_S_4_O_7_N_6_, yield, %: 85, color: brown. Found (calcd.) %: C, 47.29 (47.36); H, 3.33 (3.45); N, 10.21 (10.36); Ni, 7.08 (7.25). λ_M_ = 15 Ω^−1^ cm^−2^ mol^−1^;
(3) CuC_32_H_33_S_4_O_10.5_N_6_, yield, %: 75, color: dark brown. Found (calcd.) %: C, 47.10 (47.08); H, 3.26 (3.43); N, 10.01 (10.29); Cu, 7.59 (7.79). *λ*_M_ = 21 Ω^−1^ cm^−2^ mol^−1^.

## 3. Results and Discussion

The Schiff base ligand (HL) was prepared by the reaction of salicylaldehyde with the sulfa drug, sulfathiazole. The composition of the Schiff base ligand (Figure 1) was determined by elemental and IR spectrum analyses (Table 1). The ^1^H and ^13^C NMR spectra of the Schiff base were recorded in DMSO-d_6_. The experimental section presents ^1^H and ^13^C NMR spectral data and their assignments. The protons attached to heteroaromatic and aromatic rings, as well as the assignments of the carbon atoms in sulfathiazole, were identified in their expected region [33]. The azomethine proton and carbon signals appeared at 8.9 and 165.5 ppm, respectively; the hydroxyl proton was present in the spectrum of the Schiff base at 12.6 ppm. The presence of a number of protons and carbons in the NMR spectra of the Schiff base was in agreement with the expected CHN analyses.

The metal(II) complexes of the Schiff base were prepared in a 1:2 stoichiometric metal:ligand molar ratio using metal chlorides. The obtained complexes were air-stable and colored microcrystalline solids. They were only soluble in DMF and DMSO.

The metal complexes were non-electrolytes because their molar conductivities measured in 10^−3^ mol L^−1^ DMF at room temperature had lower values (15–21 Ω^−1^ cm^2^ mol^−1^) [34]. On the basis of the elemental analysis and physical measurements, the following molecular formulae are proposed: [ML_2_]∙nH_2_O, where L is the deprotonated Schiff base, and M(II)=Cu(II), Co(II), and Ni(II)—*n* = 1 for the Ni(II) complex; *n* = 1.5 for the Co(II) complex; and *n* = 4.5 for the Cu(II) complex.

### 3.1. Spectral Investigations of Metal Complexes

#### 3.1.1. Infrared Spectra

In order to study the formation of the Schiff base (HL) and the binding mode to the metal ions in the complexes, the IR spectra of sulfathiazole (Stz), the Schiff base, and metal complexes were comparatively studied to make the appropriate assignments. Some of the characteristic IR spectral bands of sulfathiazole, the Schiff base, and metal complexes are given in Table 1.

In the sulfathiazole spectrum, the bands of 3354 and 3321 cm^−1^ assigned to the *ν*(NH_2_) asymmetric and symmetric stretching vibrations disappeared from the Schiff base spectrum. The forming of the expected Schiff base ligand was supported by the new band at 1617 cm^−1^ that was attributed to the *ν*(C=N) azomethine group [35,36]. In the metal complex spectra, this band was shifted in the range of 1569–1577 cm^−1^, indicating the coordination of the Schiff base through the N atom of azomethine group to the central metallic ion. The deprotonation of the phenolic group was highlighted by the disappearance of the 3375 cm^−1^ band from the complex spectra, which appeared in the spectrum of the Schiff base. The band at 1274 cm^−1^ assigned to a ν(C-O) vibration was shifted by 21–36 cm^−1^ compared to the Schiff base as a consequence of the deprotonation and coordination of the phenolic oxygen [35].

Sulfonamides exhibit ν(N-H) stretching vibrations of the -SO_2_-N-H group in the range of 3320–3250 cm^−1^ [37,38], and for sulfathiazole and the spectrum of the Schiff base, it appeared at 3282 cm^−1^. The presence of this band at the same value in the spectra of the metal complexes indicated that the N atom of this group did not participate in the coordination to the central metallic ion. The *ν*(C=N) thiazole ring stretching vibration that appeared at 1540 cm^−1^ in the sulfathiazole and Schiff base spectra was shifted to lower frequencies at 1480–1485 cm^−1^ in the IR of the complexes, which means that the Schiff base was coordinated through the N thiazole atom with the central metallic ion. The bands observed at 3445 cm^−1^ in the spectra of metal complexes were assigned to some water molecules whose nature was determined by thermal studies. Some new bands in the 560–570 cm^−1^ and 450–455 cm^−1^ ranges, which were attributed to ν(M-N) and ν(M-O) stretching vibrations, were identified in the spectra of the metal complexes, considering the decrease in stretching frequency as an M–X bond becomes more ionic [39,40]. These data were in agreement with the results of molecular modelling.

Based on the IR data, the coordination of the Schiff base to metal(II) ions occurred in a tridentate mode by the nitrogen atoms of the azomethine group and of the thiazole ring and oxygen atom of the phenolic.

#### 3.1.2. Electronic Spectra and Magnetic Moments 

The electronic spectra of the Schiff base ligand and the three metal complexes were recorded in the 5000–50,000 cm^−1^ range in the solid state, and the obtained data were correlated with the magnetic moments in order to obtain information regarding the geometry of the metal complexes.

The Schiff base electronic spectrum was found to contain two absorption bands at 38,460 (260 nm) and 29,850 (335 nm) cm^−1^, which were assigned to the intra-ligand π → π * and n → π * transitions of the –C=C– (aromatic ring) and –C=N– (azomethine) groups, respectively. These bands appeared to be shifted to lower values in the complex spectra, which proved the coordination of the ligand to the metallic ions. The electronic spectrum of [CoL_2_]·1.5H_2_O contained two bands at 21,491 (465) cm^−1^ (nm) and 18,543 (539) cm^−1^ (nm), which were assigned to ^4^T_1g_(F) → ^4^A_2g_(F) and ^4^T_1g_(F) → ^4^T_1g_(P), respectively, in an octahedral geometry [41]. The value of the magnetic moment for Co(II) complex was 4.75 BM, which corresponded to an octahedral environment [42]. The spectrum of [NiL_2_]·H_2_O complex presented two bands at 23340(428) and 17625(567) cm^−1^(nm) that were assignable to the ^3^A_2g_(F) → ^3^T_1g_(P) and ^3^A_2g_(F) → ^3^T_1g_(F) transitions, respectively, which are characteristic of Ni(II) complexes with octahedral geometry [30,31,32,33,34,35,36,37,38,39,40,41,42,43]. This geometry around the Ni(II) ion was also supported by the magnetic moment value of 2.97 BM. The Cu(II) complex showed bands at 19,180 (521) and 14,985 (667) cm^−1^(nm) that could be attributed to the ^2^B_1g_ → ^2^E_g_ (d_xz, yz_ → d_x^2^-y^2^_) and ^2^B_1g_ → ^2^A_1g_ (d_xy_ → d_x^2^_-_y^2^_) transitions, respectively. The broad band from 14,985 cm^−1^ indicated that copper (II) ion has a tetragonally distorted octahedral geometry [38,41]. This is due to the Jahn Teller effect that appears on the d^9^ electronic ground state of hexacoordinated system, along with the three different donor atoms: N-azomethine, N-thiazole, and O-phenolic. Thus, the elongating of one *trans* pair of coordinate bonds and the shortening of the remaining four occur [43]. The magnetic moment for Cu(II) complex was 1.76 BM, which indicated the spin coupling absence [44].

### 3.2. Thermal Behaviour of the Complexes

**Figure 2:**
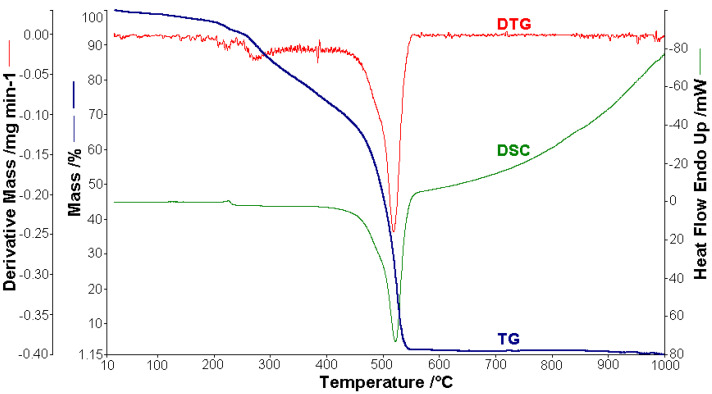
Thermoanalytical curves of [CoL_2_]·1.5H_2_O.

**Figure 3:**
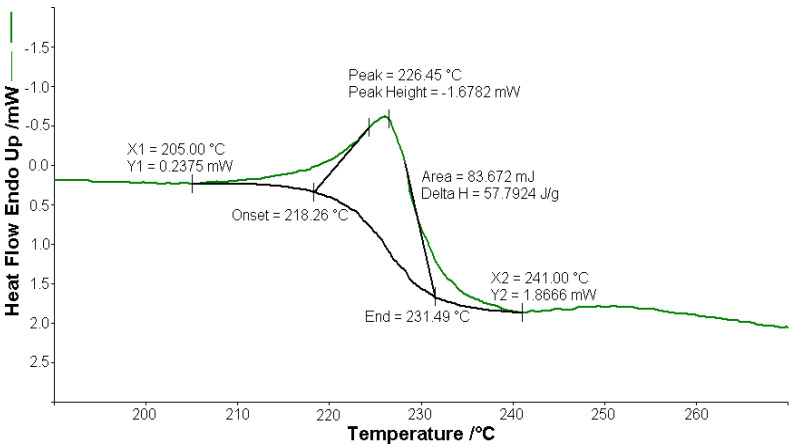
Endothermic peak (∆*H* = 57.79 J g^−1^) on the DSC curve at 226.4 °C for [CoL_2_]·1.5H_2_O.

**Figure 4:**
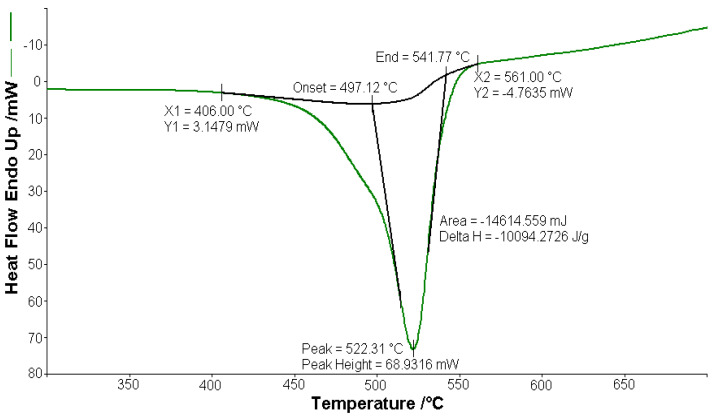
Exothermic peak (∆*H* = −10,094.2 J g^−1^) on the DSC curve at 522.31 °C for [CoL_2_]·1.5H_2_O.

**Figure 5:**
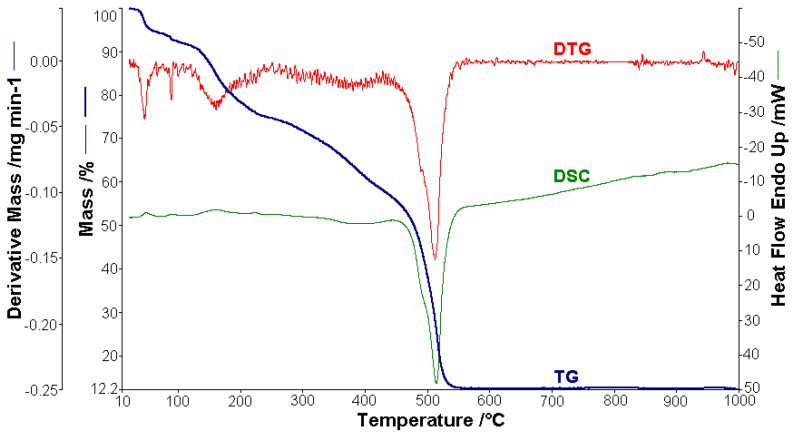
Thermoanalytical curves of [NiL_2_]·H_2_O.

**Figure 6:**
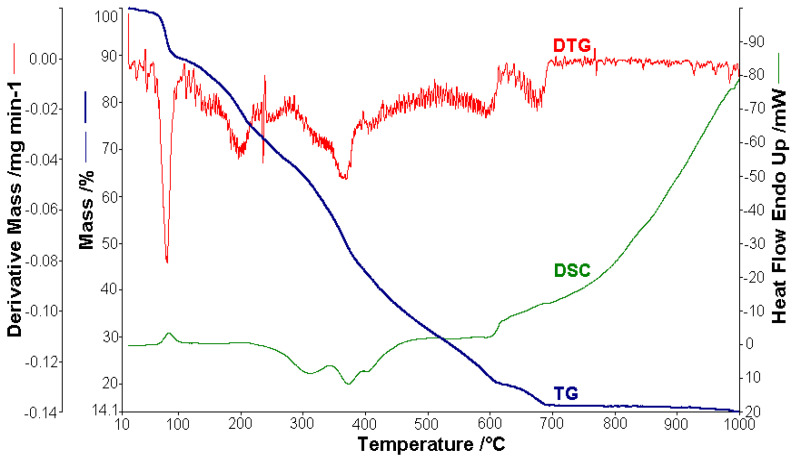
Thermoanalytical curves of [CuL_2_]·4.5H_2_O.

Thermal analysis studies of metal–organic complexes are generally required before further physical–chemical investigations and prior to their employment as useful materials [21,22,23,24,25,26,27,28]. Thermogravimetric analysis was performed to confirm the composition of the complexes and to give information for their thermal stability. The thermal analysis curves (TG, DTG, and DSC) of the metal complexes are shown in Figure 2, Figure 3, Figure 4, Figure 5 and Figure 6. During heating from room temperature up to 1000 °C in air, the three investigated complexes underwent a complete decomposition with a remaining black residue. A final residue that corresponded to the metal oxide or a mixture of metal oxide and carbonaceous matter was also proved by chemical analysis.

#### 3.2.1. Thermal Analysis of (1)

A sample of 1.448 mg of [CoL_2_]·1.5H_2_O underwent thermal decomposition in the range from RT to 1000 °C at 10 K min^−1^ in a dynamic air atmosphere. Figure 2 presents the decomposition process. In the first stage, between 23 and 205 °C with a weakly endothermic effect, the mass variation (Δ*m*_exp_ = 3.49%) corresponded to a loss of 1.5 lattice water moles (Δ*m*_calc_ = 3.36%). The second stage of decomposition occurring in the temperature range of 205–242 °C with a Δ*m*_exp_ = 9.89% might be interpreted as the loss of one ammonia molecule (Δ*m*_calc_ = 2.14%), with an endothermic peak on the DSC curve at 226.4 °C and an absorption of heat ∆*H* = 57.79 J g^−1^ (Figure 3). The third stage between 242 and 1000 °C with a mass loss of Δ*m*_exp_ = 92.56% and an exothermic effect with a peak on the DSC curve at 522.31 °C corresponded to the loss of organic moieties (Δ*m*_calc_ = 92.65%). The strong heat release ∆*H* = 10,094.2 J g^−1^ (Figure 4), which characterized the transformation, indicated that the combustion of the organic fragments also carried along parts of the central metallic ion as products that resulted from the breaking of all metal–ligand bonds [45]. The obtained residue mass was 1/4CoO (∆*m*_exp_ = 1.14% and ∆*m*_calc_ = 1.75%).

#### 3.2.2. Thermal Analysis of (2)

The curves that describe the thermal behavior of the [NiL_2_]·H_2_O complex are presented in Figure 5. The analysis was carried out using a 1.252 mg metal complex. The mass variation Δ*m*_exp_ = 5.36% in the range of 22–62 °C was attributed to the release of humidity water. The process is characterized by an endothermic peak (∆*H* = 75.93 Jg^−1^) on the DSC curve. One molecule of lattice water was lost in the range from 62 to 102 °C (∆*m*_exp_ = 2.52%; ∆*m*_calc_ = 2.12%), with a weakly endothermic peak (∆*H* = 40.92 Jg^−1^) on the DSC curve at 90.2 °C [46]. Then, the complex compound gradually decomposed to the corresponding metallic oxide residue at a higher temperature. Therefore, in the next stage (106–560 °C), the lost mass (∆*m*_exp_ = 75.22%; ∆*m*_calc_ = 75.81%) corresponded to the loss of the organic moieties when the thermal decomposition had a strong exothermic effect on the DSC curve with a peak at 514.4 °C and an enthalpy decrease (∆*H* = −6420.7 Jg^−1^). The final black residue was a mixture of NiO and 4C (∆*m*_exp_ = 16.89%; ∆*m*_calc_ = 16.64%).

#### 3.2.3. Thermal Analysis of (3)

In the case of [CuL_2_]·4.5H_2_O, 1.512 mg were used for thermal analysis (Figure 6). The observed mass variation Δ*m*_exp_ = 1.09% in the range of 20–60 °C with a weakly endothermic effect corresponded to the release of humidity water. From 60 to 110 °C, 4.5 molecules of lattice water were lost, with an endothermic peak (∆*H* = 176.37 J g^−1^) on the DSC curve at 84.4 °C (Δ*m*_exp_ = 9.89%; ∆*m*_calc_ = 9.41%). In the next stage (110–700 °C), the lost mass (∆*m*_exp_ = 74.40%; ∆*m*_calc_ = 74.78%) corresponded the loss of the organic moieties and was characterized by an exothermic effect with two peaks on the DSC curve at 371.2 °C (∆*H* = −428.25 Jg^−1^) and 403.1 °C (∆*H* = −7.11 J g^−1^), which proved the complexity of the thermal decomposition, leaving CuO and 4C as a residue (∆*m*_exp_ = 14.12%; ∆*m*_calc_ = 14.86%).

The results obtained from thermogravimetric analysis, which are presented in Table 2, validated the formulas resulting from the analytical data. Different numbers of decomposition steps were evidenced depending on the metal type. Moreover, the Co(II) complex proved to be the most stable against decomposition under air. The sublimation of cobalt compounds both in inert and oxidative atmospheres was also evidenced in an early study [45].

### 3.3. XRD/EDS/SEM Analyses

**Figure 7:**
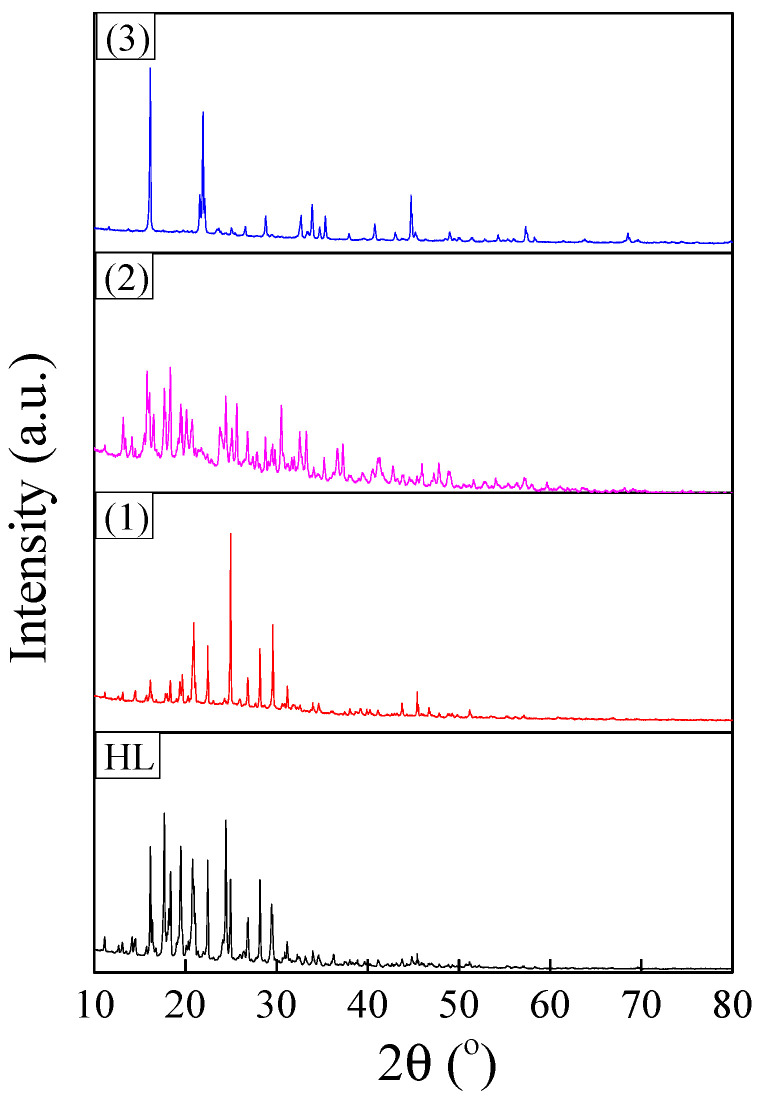
XRD patterns of polycrystalline powders corresponding to ligand and complexes.

XRD patterns of synthesized ligand and complexes are shown in Figure 7. The reflections were indexed to a monoclinic crystalline structure (space group *P2*_1_/*c*) for all investigated samples [47]. The FullProf software was used to evaluate the structural parameters of the polycrystalline powders by pattern matching. The refinement results are summarized in Table 3. The unit cell volume of the complexes increased in the order of (2) < (1) < (3). This variation in unit cell parameters was in agreement with molecular modelling results and was determined by structural changes induced by the Jahn Teller effect. This effect increased in the same order, being the strongest for the copper complex.

EDS analysis allowed for the identification and quantification of nitrogen, sulphur, and metals from the ligand (Supplementary Materials Figure S1) and complexes. The contributions of nitrogen, sulphur, and metals in at. % in the polycrystalline powders are presented in Table 4. It can be noticed that the N:S and N:M(II) atomic ratios were close to the theoretical ones (1.5 and 6.0, respectively), with the largest deviation from the estimated values being obtained for the nickel complex (Table 4).

SEM images revealed the morphology of the polycrystalline powders. Hard agglomerates with micrometer-range sizes were observed for the Schiff base and complexes (Supplementary Materials Figure S2).

### 3.4. Molecular Modelling of the Schiff Base Ligand

**Figure 8:**
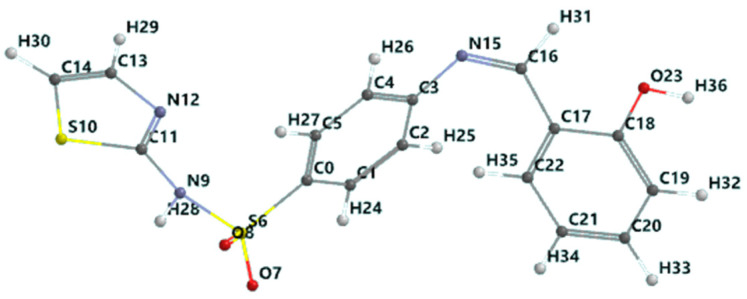
Schiff base optimized geometry in the singlet ground state (color codes are white: H; red: O; blue: N; black: C; and yellow: S).

The chelating behavior and biological activity of the Schiff base derived from sulfathiazole and salicylaldehyde are strongly correlated with its structural, electronic, and energetic properties. Therefore, the prediction of these properties would provide the elements needed to elucidate the covalent and non-covalent interactions in which this ligand participates. The estimation of the quantitative properties was performed by ab initio quantum-mechanical calculations at the level of approximation of DFT carried out in a Spartan molecular modelling environment, with the input being the Schiff base molecule geometry that corresponded to the local minimum on a multidimensional surface (hypersurface) of potential energy. In Figure 8, the optimized geometry of the Schiff base molecule in the singlet ground state is presented.

**Figure 9:**
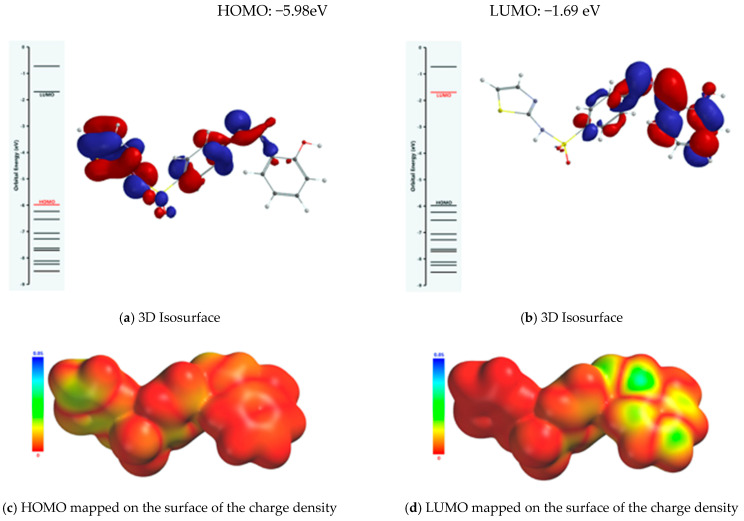
Frontier molecular orbitals (FMOs) of the Schiff base ligand.

The property of the Schiff base to form stable complexes with different transition metals derives from the existence of atoms in the vicinity of which the electron density is high, as they can coordinate the transition metal; covalent interactions between the ligand and the metallic ion are established through these atoms while simultaneously partially sharing the positive charge of the metallic ion. The mechanism of chelation was also analyzed from the perspective of the frontier molecular orbital theory HOMO (highest occupied molecular orbital) and LUMO (lowest unoccupied molecular orbital), as donor–acceptor interactions between the ligand and the metallic ion are mediated by peripheral molecular orbitals—the HOMO belonging to the ligand donates energized electrons to the LUMO of the metallic ion. From the analysis of the isosurface of the highest occupied molecular orbital and its mapping on the surface of the total charge density (Figure 9), it turned out that there are three metallic ion binding centers: the oxygen atom of the phenolic group and the two nitrogen atoms of the azomethine group and thiazolic ring.

**Figure 10:**
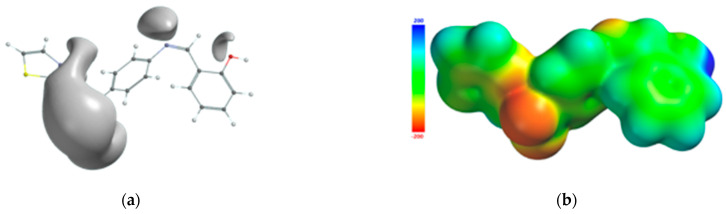
Isosurface of the molecular electrostatic potential of the Schiff base ligand (**a**); molecular electrostatic potential mapped on the surface of the total charge density (**b**).

**Figure 11:**
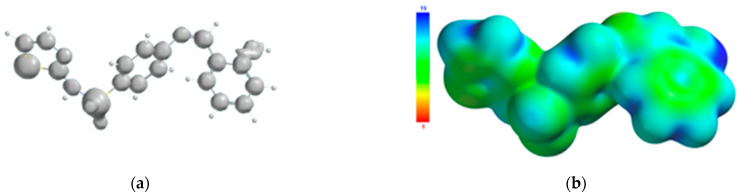
Isosurface of the ionization potential of the Schiff base ligand (**a**); ionization potential mapped on the surface of the total charge density (**b**).

The atoms of the Schiff base involved in the coordination of the transition metal were also identified from the 3D graphical model of the effective distribution of the electrons in the ligand molecule. This was obtained by mapping the molecular electrostatic potential on an isodensity surface of 0.002 electrons/a.u.^3^, which approximated the van der Waals radius of the ligand molecule (Figure 10). The 3D map of the spatial distribution of the electrons in the ligand molecule highlighted the following: (a) the size of the ligand molecule and its shape that excludes steric hindrances determine the coordination of the transition metal with the formation of an octahedral geometry, and (b) the reactive centers on the molecular surface correspond to the more negative values of the molecular electrostatic potential and are adjacent to the oxygen atom of the phenolic group and to the nitrogen atoms of the azomethine group and the thiazole ring. The atoms in the ligand molecule participating in the covalent interactions with the metal ion were also confirmed by the 3D graphical model obtained by mapping the ionization potential on the surface of the total electric charge density (Figure 11).

**Figure 12:**
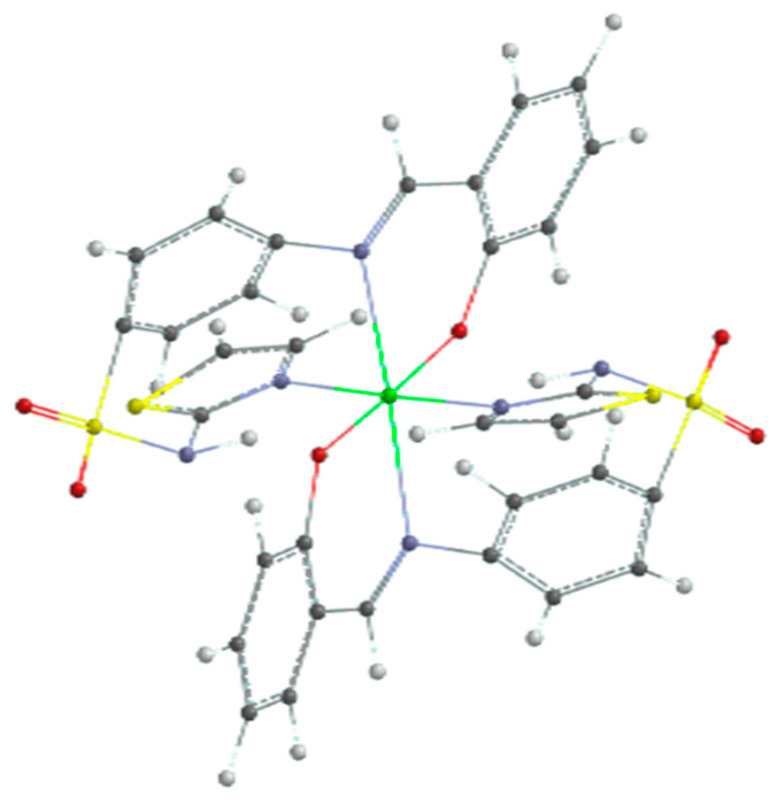
Optimized geometry of copper(II) complex (color codes are white: H; red: O; blue: N; black: C; yellow: S; and green: Cu).

The results of the quantum calculation of the electronic structure of the ligand corroborated with the experimental data led to the hypothesis of an octahedral geometry of the ML_2_ complex. The result of the geometric optimization calculation of the CuL_2_ complex is shown in Figure 12.

The calculations of quantum descriptors of the Schiff base (HL) and copper (II) complex reactivity are given in Table 5. The parameters of energy gap, Δ*E* = *E*_LUMO_ − *E*_HOMO_, absolute electronegativity (*χ*), absolute hardness (*η*), global softness (*S*), chemical potential (μ), and electrophilicity index (*ω*) were calculated according to the literature data [48,49,50,51,52] and with the following formulas:

$$\chi =-\frac{E_{\mathrm{LUMO}}+E_{\mathrm{HOMO}}}{2}\chi =-\frac{E_{\mathrm{LUMO}}+E_{\mathrm{HOMO}}}{2}$$

$$\mu =-\chi \;S=\frac{1}{2\eta }\omega =\frac{\mu^{2}}{2\eta }=\frac{\chi^{2}}{2\eta }$$

Quantum-mechanical calculations have another molecular parameter: the electric dipole moment, which indicates the partial separation of the electric charges in a molecule and it is also a predictor of the chemical reactivity of a molecule. The magnitude of the dipole moment vector of 4.99 D confirmed the good docking capacity of the Schiff base to the surface of its biological receptor.

### 3.5. Antibacterial Activity

A major problem in public health is the resistance of new bacterial strains to current antibiotics. Thus, it is necessary to find alternative compounds that behave as drugs. Over the past few years, researchers have studied the synthesis of new metal complexes with new organic ligands and tested them for antimicrobial activity.

In this work, we tested the synthesized compounds for in vitro antibacterial activity against two Gram-negative (*E. coli* and *P. aeruginosa*) and two Gram-positive (*S. aureus* and *B. subtilis*) clinical isolate bacterial strains by measuring the size of the bacteriostatic diameter (Table 6). The obtained results were compared with those of the commercial drug amoxicillin, which was used as standard. The solvent DMSO alone does not show any antibacterial effects. At this time, we have no data on antibacterial activity using metal chlorides as substrates on the bacteria studied in this paper.

The results indicated that complexes (1), (2), and (3) were all better than HL in antibacterial action, and their activity was in the order of (3) > (1) > (2). There are two theories that can explain this increased activity of complexes: (1) the overtone concept [53], which considers that the compound solubility in the cell membrane has an important role in antibacterial activity because it allows for the passage of only soluble materials in lipids, and (2) Tweedy’s chelation theory [54], which explains that the polarity of a metal ion is highly reduced because of the ligand orbital overlap and the positive charge division of the central metallic ion with the donor atoms of the ligand.

**Figure 13:**
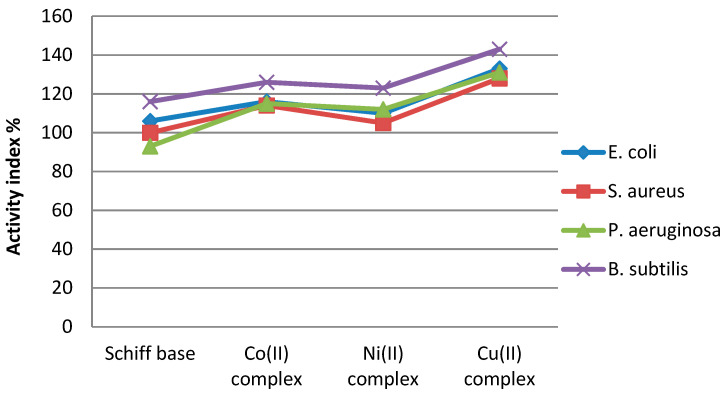
Activity index values of compounds.

Activity index values of complex compounds were calculated according to the following relation: AI = ((Inhibition zone of compound/Inhibition zone of standard)) × 100. The results presented in Figure 13 indicate that the Cu complex showed the highest activity index.

## 4. Conclusions

Three new metal(II) complexes with the Schiff base derived from sulfathiazole and salicylaldehyde were obtained in a molar ratio M(II):L of 1:2. IR data showed that the Schiff base behaved as a tridentate, monoanionic NNO-chelating agent with azomethine-N, thiazole-N, and phenolic-O atoms. The metal complexes were found to be non-electrolytes with a molar conductivity in the range of 15–21 Ω^−1^ cm^2^ mol^−1^. Electronic spectra indicated octahedral geometry for the Co(II) and Ni(II) complexes and a tetragonally distorted octahedral geometry for the Cu(II) complex. The values of magnetic moments were determined.

The results from the thermal analysis confirmed their composition and thermal stability. XRD analysis evidenced the monoclinic structure for all the investigated powders. Moreover, the unit cell volume of the complexes was found to increase in the order of (2) < (1) < (3). Hard agglomerates with micrometer-range sizes were evidenced by SEM/EDS analysis, with the N:S and N:M(II) atomic ratios close to the theoretical values (1.5 and 6.0, respectively). The DFT calculation of the electronic structure of the Schiff base indicated the existence of three metal ion coordination sites that are energetically and sterically favorable for the formation of two chelating rings with 6 and 10 atoms, respectively. The values of the chemical reactivity descriptors derived from the DFT formalism showed a higher reactivity of the metal complex with respect to the ligand; this is a consequence of a fine adjustment of the electronic and steric properties induced by the covalent interactions between the Schiff base donor atoms and the metallic ion. 

The Schiff base and its metal complexes were tested against the *E. coli*, *P. aeruginosa*, *S. aureus*, and *B. subtilis* strains, and their inhibitory effects on the growth of bacterial strains varied in the following order of (3) > (1) > (2).

## Figures and Tables

**Table 1:** Assignments of the vibrational spectra (frequency: *ν*, cm^−1^) of sulfathiazole, the Schiff base, and metal complexes.

Stz	HL	(1)	(2)	(3)	Assignments
-	-	3445	3445	3445	νH_2_O
-	3375	-	-	-	ν(OH) phenolic
3354	-	-	-	-	ν(NH_2_) asym
3321	-	-	-	-	ν(NH_2_) sym
3282	3282	3282	3282	3282	ν(SO_2_ –NH)
-	1617	1577	1569	1569	ν(C=N) azm
1540	1540	1485	1480	1485	ν(C=N) thiazole ring
1345	1345	1345	1345	1345	ν(SO_2_) asym
-	1274	1295	1305	1310	ν(C-O) phenolic
1110	1110	1110	1110	1110	ν(SO_2_) sym
635	635	635	635	635	ν(C-S) thiazole ring
-	-	560	570	565	ν(M-N)
-	-	450	450	455	ν(M-O)

**Table 2:** Thermal behavior of metal complexes.

Stage	Metal Complex	*Temp. Range*/°C	DSC Parameters	∆*m_exp_*/%	∆*m_calc_*/%	Assignment
			∆*H*/Jg^−1^; *T_max_*/°C			
**[CoL_2_]·1.5H_2_O**						
1	CoC_32_ H_27_S_4_O_7.5_N_6_	23–205	Weakly endothermic	3.49	3.36	Loss of lattice water molecule
	↓-1.5H_2_O					
2	CoC_32_ H_24_S_4_O_6_N_6_	205–242	57.79; 226.4	2.68	2.14	Loss of ammonia molecule
	↓-NH_3_					
3	CoC_32_ H_21_S_4_O_6_N_5_	242–1000	−10094.2; 522.31	92.56	92.65	Loss of organic moieties and a part of Co(II)
	↓-organic moieties1/4Co			1.14	1.75	residue (1/4CoO)
**[NiL_2_ ]·H_2_O**						
1	NiC_32_ H_26_ S_4_O_7_N_6_	22–62	75.93; 47.9	5.36	-	Water humidity
	↓-humidity					
2	NiC_32_ H_26_S_4_O_7_N_6_	62–102	40.92; 90.2	2.52	2.12	Loss of lattice water molecule
	↓-H_2_O					
3	NiC_32_H_24_ S_4_O_6_N_6_	106–560	−6420.7; 514.4	75.22	75.81	Loss of organic moieties
	↓-organic moieties NiO and 4C			16.89	16.64	Residue (NiO and 4C)
**[CuL_2_ ]·4.5H_2_O**						
1	CuC_32_ H_33_ S_4_O_10.5_N_6_	20–60	Weakly endothermic	1.09	-	Water humidity
	↓-humidity					
2	CuC_32_ H_33_S_4_O_10.5_N_6_	60–110	176.37; 84.4	9.89	9.41	Loss of lattice water molecules
	↓-4.5H_2_O					
3	CuC_32_ H_24_ S_4_O_6_N_6_	110–700	−428.25; 371.2	74.40	74.78	Loss of organic moieties
	↓-organic moietiesCuO and 4C		−7.11; 403.1	14.12	14.86	Residue (CuO and 4C)

**Table 3:** Structural parameters of the Schiff base (HL) and metal complexes (**1**–**3**).

Parameter	(HL)	(1)	(2)	(3)
*a*/Å	9.22 (1)	9.29 (1)	9.29 (9)	9.34 (9)
*b*/Å	11.34 (1)	11.39 (6)	11.44 (1)	11.45 (3)
*c*/Å	28.41 (7)	28.64 (5)	28.46 (2)	28.56 (3)
*α*/°	90.00	90.00	90.00	90.00
*β*/°	91.8 (3)	92.1 (4)	92.4 (1)	92.0 (3)
*γ*/°	90.00	90.00	90.00	90.00
*V*/Å^3^	2970.0 (8)	3030.8 (4)	3025.5 (9)	3056.6 (7)

**Table 4:** Elemental composition of the investigated Schiff base (HL) and metal complexes (from EDS analysis).

Compound	N, % at	S, % at	Co, % at	Cu, % at	Ni, % at	N:S	N:M(II)
HL	11.0	7.2	-	-	-	1.52	-
(1)	9.2	6.0	1.5	-	-	1.53	6.1
(2)	3.5	2.4	-	-	0.7	1.45	5.0
(3)	6.1	4.0	-	1.1	-	1.52	5.5

**Table 5:** The calculated quantum chemical parameters of the Schiff base (HL) and Cu(II) complex.

Compound	*E*_HOMO_ /eV	*E*_LUMO_ /eV	Δ*E* /eV	*χ* /eV	*μ* /eV	*η* /eV	*S* /eV^−1^	*ω* /eV
HL	−5.98	−1.69	4.29	3.84	−3.84	2.14	0.23	3.43
Cu(II) complex	−5.90	−2.65	3.25	4.28	−4.28	1.62	0.31	5.62

**Table 6:** The inhibition diameter zone (mm) of the Schiff base and metal complexes against bacterial strains.

Compound	*E. coli*	*S. aureus*	*P. aeruginosa*	*B. subtilis*
HL	32	35	30	35
(1)	35	40	37	38
(2)	33	37	36	37
(3)	40	45	42	43
Amoxicillin	30	35	32	30
DMSO	-	-	-	-

## Data Availability

Data is contained within the Supplementary Material.

## Supplementary Materials

The following are available online. Figure S1: EDS spectrum of Schiff base ligand; Figure S2: SEM images of (a) Schiff base; (b) [CoL_2_]∙1.5H_2_O; (c) [CuL_2_]∙4.5H_2_O; (d) [NiL_2_]∙H_2_O.

## Sample Availability

Samples of the compounds are not available from the authors.
