# Supplementary material for: Bioactive Co(II), Ni(II), and Cu(II) Complexes Containing a Tridentate Sulfathiazole-Based (ONN) Schiff Base
Source: Molecules. 2021 May 20;26(10):3062. doi: 10.3390/molecules26103062 (PMC8161335; doi:10.3390/molecules26103062)
Supplement: Supplementary file 1 [file molecules-26-03062-s001.zip › molecules-1199494-supplementary.pdf]

SUPPLEMENTARY MATERIALS

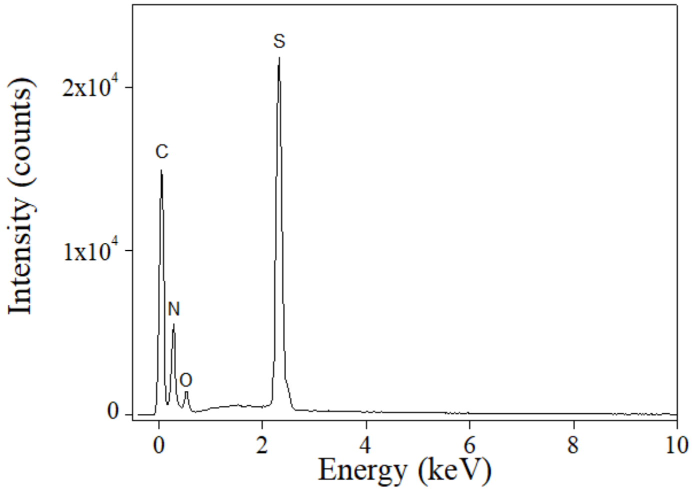

Figure S1 EDS spectrum of Schiff base ligand

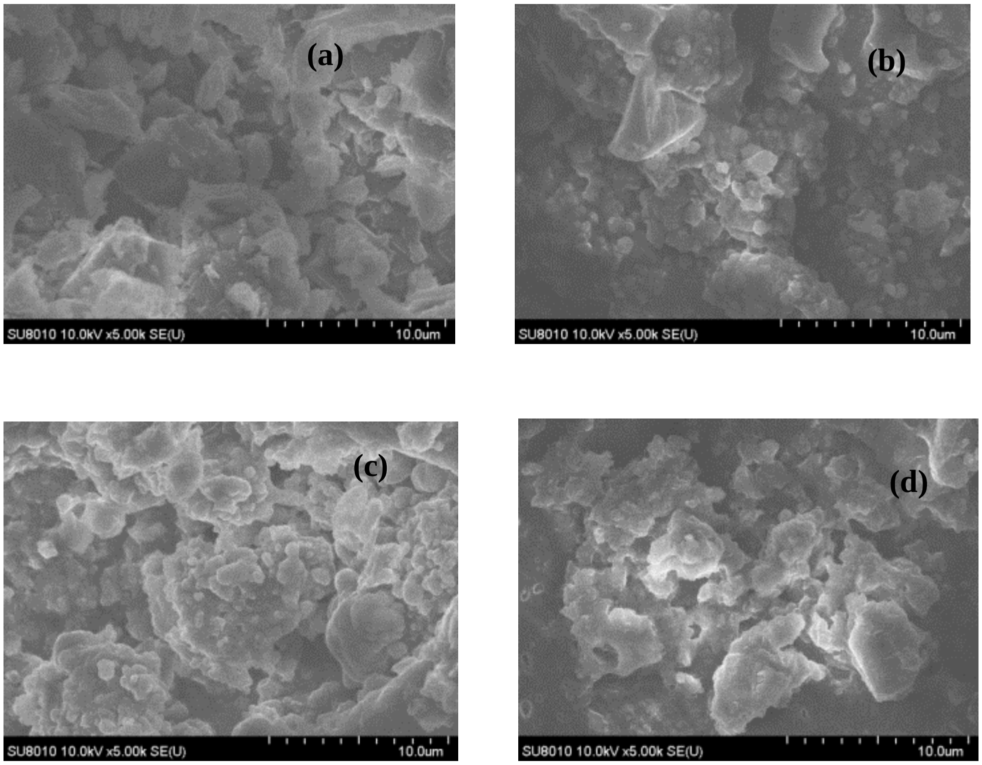

Figure S2 SEM images of (**a**) Schiff base; (**b**) $[CoL_2]\cdot1.5H_2O$; (**c**) $[CuL_2]\cdot4.5H_2O$; (**d**) $[NiL_2]\cdot H_2O$
